# Process and Outcome of Community Engagement Event on Substance Use and Addiction Risks Facing Their Immigrant Communities in Regina, Saskatchewan

**DOI:** 10.1177/11782218221150109

**Published:** 2023-01-23

**Authors:** Geoffrey Maina, Ghazal Mousavian, Jordan Sherstobitoff, Rejina Kamrul, Barbara Twum-Antwi, Kennedy Lewis, Francia Malonga, Thea Herzog, Razawa Maroof, Denis Okinyo-Owiti

**Affiliations:** 1 College of Medicine, University of Saskatchewan, Regina Campus, SK, Canada; 2 InterDisciplinary Studies, University of Saskatchewan, Prince Albert, SK, Canada

**Keywords:** Immigrants, substance use, community-based research, knowledge translation, community mobilization

## Abstract

Canada is a significant destination for immigrants who are drawn from different ethnic and cultural backgrounds some of whom have a hidden risk for substance use disorders due to acculturation stress and are not screened for risks of substance use or addiction when considering medical admissibility. Not surprisingly, healthcare providers in Regina are reporting a noticeable increase in substance use among immigrants. These immigrants experience barriers in seeking substance use prevention and treatment services due to diverse challenges: stigma, shame, and lack of knowledge of existing services. Considering the discussed challenges and risks of substance use disorders in immigrant communities, creating a safe space for discussing these topics is urgent. To understand and address these challenges, a connection grant from the Saskatchewan Health Research Foundation (SHRF) to mobilize immigrant communities in Regina to explore substance use issues and their impact on the community was sought and received. Subsequently, a *Zoom* knowledge-sharing event brought settlement agency stakeholders together to deliberate issues on substance use and addiction faced by immigrants in Regina, Saskatchewan. The *Zoom* session included presentations on immigrants and substance use from the clinical, community, and lived experience perspectives of immigrants. Because of the challenges and risks, this community consultation process revealed that acculturation stress and the ease of obtaining socially acceptable substances fuel substance use and addiction among immigrants in Regina; this is further exacerbated by the lack of programming available to prevent and reduce the risks of substance use in this population. A team of knowledge keepers with lived experiences, service providers, and researchers was assembled to explore substance use and addiction among immigrants. This manuscript reports the process of community engagement to identify solutions to this budding issue. The strengths, challenges, and lessons learned are identified.

## Background

Canada has the eighth-largest immigrant population globally, with those foreign-born making up about one-fifth of the entire population.^1^ Immigrants drawn to Canada from different nationalities and cultural identities, each have their own stories and motivations for migration.^2,3^ In 2019, the top 10 countries of origin for immigrants to Canada were India (85 585), China (30 260), the Philippines (27 815), Nigeria (12 595), the US (10 800), Pakistan (10 790), Syria (10 120), Eritrea (7025), Korea (6110), and Iran (6055).^4^

Immigrants are screened for medical conditions that may pose a public health risk or burden to the Canadian healthcare system; these include HIV, T.B., and syphilis.^5,6^ While this screening ensures that only healthy immigrants are admitted to Canada, the system does not screen for invisible conditions, conditions such as trauma, current substance use, and mental illnesses, which have the propensity to increase the risk of substance use in any population. Upon arrival, immigrants often face challenges regarding language barriers, healthcare access, isolation, and cultural differences.^7^ Additionally, compared to Canadian-born individuals, immigrants are more likely to be underemployed/unemployed, work in low-skilled occupations, and earn lower wages, even if they possess a superior level of education.^8^ These challenges contribute to the stressful process of immigration and settlement and provide insight into the downturn in health indicators, including increased risks for substance use, mental illness, and other stress-related conditions that immigrants face.^9^

Epidemiologic studies suggest a low prevalence of substance use among migrant populations compared to the general population.^10,11^ Nevertheless, some immigrants may have had prior exposure to different addictive substances before they immigrate, some of which are unavailable or uncommon in Canada. For instance, khat is used in parts of Africa (notably in Somalia), and the Middle East but is not available in Canada.^12,13^ Such immigrants may seek alternatives to replace these substances. Moreover, different cultural backgrounds influence attitudes and beliefs about substance use and misuse. For example, subcultures with a historical tradition of using plant products for ceremonial use can influence contemporary cannabis use.^14^

At the time of immigration, immigrants may be oblivious to the Canadian substance use landscape. Settlement agencies provide information and support to enable them to settle and integrate into Canadian life. Still, they do not provide information on the risks of substance use and addiction that immigrants may encounter. For instance, immigrants are confronted with the reality of ease of acceptability and accessibility of alcohol and marijuana, 2 of the most widely used psychoactive drugs used by 76% and 21% of Canadians, respectively.^15^ Moreover, Canada has the second-highest per capita use of prescription opioids globally; in 2018, 9.6% were used for non-medical purposes.^16,17^ The harms associated with opioid misuse may be due to illicit drugs such as fentanyl, but also from opioid prescriptions.^18^

The ease at which alcohol and marijuana can be accessed in Canada, combined with pressure to acculturate, leaves immigrants without prior exposure especially vulnerable to using these drugs socially and/or recreationally.^19^ The risk of substance use among immigrants in Canada varies by place of origin and increases with the length of time in Canada, and although their Canadian-born counterparts have higher alcohol and marijuana use, it is still important to monitor these rates amongst immigrants.^20,21^

In immigrant communities, the individual and the family suffer in silence when one develops an addiction; this is attributable to stigma, shame, or fear of “losing face” in the community. The issue is further exacerbated when affected members lack the knowledge and/or the resources necessary to support recovery and/or treatment for addiction.^22^ Another way these communities may find themselves unable to receive and/or locate these resources is due to cultural inaccessibility; for cultural and religious reasons, it is expected in some countries for meetings to be gender segregated. Therefore, some immigrants may find support groups such as Alcoholics Anonymous unsuitable or even uncomfortable, which adds challenges to the issue. Immigrants may feel that disclosing family struggles relating to addiction, such as in the setting of counseling or a support group, is a betrayal of loyalty and honor. Additionally, 70% of immigrants report a language other than English or French as their mother tongue, so they may lack the necessary English language proficiency to effectively utilize the existing services.^23,24^

The limited research on immigrants on substance use has focused on how it intersects with mental illness and the disparity in substance use when comparing those born in Canada.^25-27^ Much of what is currently known about substance use amongst immigrants have been drawn from studies conducted primarily in Ontario, which has a significantly greater population with greater cultural diversity than Saskatchewan^28-30^

Despite past studies on substance use and addiction among immigrants, little information is available on the substance use of migrant populations compared with the general population in Canada, the association between substance use, socioeconomic status, and pre-and post-immigration migration factors.^31^ Addiction issues may not be addressed in immigrant families and communities without empowerment due to the socio-cultural norms that demand silence and secrecy. This demonstrates the urgency to innovatively motivate immigrant communities in a culturally acceptable manner; doing so enables them to discuss and act on substance use in their community and assists in making them feel comfortable throughout their involvement.

A recent collaborative meeting for a project led by Pandey et al^32^ that focuses on the barriers immigrants in Regina face brought about concern regarding an increasing number of immigrants developing substance use disorders. Collaborating partners, including stakeholders in Regina’s immigrant settlement and service industry, noted that stress associated with immigration and settlement appears to be responsible for some of the maladaptive coping behaviors of immigrants. Anecdotal reports suggest that the nature and trend of substance use differ amongst family members, with alcohol being the predominant substance of abuse for parents. At the same time, newcomer youth prefer to use e-cigarettes, alcohol, and marijuana.^33^ There also appears to be a distinction in motivation for substance use in immigrant families, with parents being driven primarily by acculturation stress and youth being driven by peer pressure and a need to belong.^34^ Substance use among immigrants is further aggravated by the availability and accessibility of alcohol and marijuana, 2 socially acceptable substances in Canada.^19^ Moreover, immigrants can readily access these substances in Canada regardless of gender, class, and socioeconomic status.

The population of immigrants in Saskatchewan increased from 3.6% of the total population in 2006 to 10.8% in 2016, and it continues to grow. The Provincial government has projected a population increase of 300 000 over 10 years, primarily through immigration.^35^ Given the acculturation stress that immigrants experience, which may increase the risk of substance use in immigrants, it is imperative to understand the community’s understanding and experiences with substances. This focus is of great importance, as the small population size in the province increases the likelihood that they will be overlooked in the policy and program developments employed to mitigate these risks. This is a concern because this population has been overlooked similarly in the past; for instance, the Saskatchewan Mental Health Action Plan recognizes response to diversities as one of its pillars, yet there are no known mental health and addiction programs that seek to cater to the needs of ethnic minorities in the province.^36^ This segment of society, no matter how much of the overall population it comprises, needs a safe space to talk about substance use, addiction issues, and the trends affecting them. To address them adequately, collaboration is necessary, and overlooking a population that would benefit from being involved, seen, and educated is highly counterproductive.

This paper reports a consultative process in Regina to explore substance use and addiction issues with immigrants and their communities and to explore the response. It describes the steps the research team took to conceptualize the idea, apply for seed funding to engage the immigrant community, the results of community engagement, and finally, the application for a Solutions grant.

## Applying For and Receiving a Seed Grant

Regina Immigrant Women’s Centre and Regina Community Clinic are 2 stakeholder agencies collaborating to provide services to immigrants and refugees in Regina, Saskatchewan. Regina Immigrant Women’s Centre provides settlement advising, family support, education and training, employment programs, and community outreach to immigrant and refugee women and their families. At the same time, the Regina Community Clinic is a cooperative primary health service that provides care to all backgrounds, including immigrants and government-sponsored refugees. The Open-Door Society is another immigrant settlement agency which provides interpretation services when accessing resources, such as Regina Community Clinic. Collaboration with these agencies identified the need to address substance use and addiction among immigrants. This concern was apparent to agencies and healthcare workers but invisible to the public due to the stigma associated with substance use. When an opportunity arose to apply for the seed grant called Start grant focused on addictions in 2021, a consultative meeting with immigration and settlement practitioners was arranged to further deliberate on this issue. Collaboratively, these agencies and researchers aim to impact immigrant health.^32^

Once the funding was awarded, additional consultative meetings were held to plan the knowledge-sharing event. In addition to agreeing on the agenda for the meeting, a list of potential invitees was drawn, and tasks were allocated for inviting and following up with the invitees. A poster containing the event’s details, titled “*Creating space for immigrant communities to build solutions to address substance use and addiction*,” was created. In this session, we recruited the following individuals for the study: (a) immigrants living in Regina; (b) immigrants affected by addiction, that is, individuals in recovery or their relatives; and (c) services providers in the immigration and settlement industry.

## The Knowledge-Sharing Event

The knowledge-sharing event was set for October 2, 2021, with 22 people attending via *Zoom*, including 3 research team members, 5 healthcare providers, 11 settlement workers, and 2 people with lived experiences. The session began with self-introductions and was followed by presentations regarding immigrants and substance use from the clinical, community, and lived experience perspectives. Next, an open forum was utilized to discuss the substance use priorities for the immigrant population in Regina. Notes from the forum, including the chat messages, were recorded, compiled, and then summarized in Table 1.

**Table 1.** Summary of the main points generated from the consultation meeting from the perspective of immigrant stakeholders (academic, health providers, and settlement service providers).

Issues and trends	Proposed solutions
Immigrants as a heterogeneous socio-cultural group have varying risks for substance use based on socio-cultural and pre- and post-immigration experiences.	• Understand the risk profile for each community
	• Identify and involve the community leaders and opinion shapers for each ethnic group address associated with substance use in the community.
	• Create awareness about the impact of substance use on family and community
	• Build capacity for community leaders to drive substance use interventions.
	• Support systems for parents to build capacity, especially for community organizations to support families struggling with addiction.
	• When discussing a sensitive topic such as substance use and addiction in the community, it should be handled with sensitivity due to cultural dynamics.
	• Conversations about substance use should not be judgmental and should focus on the positives to prevent alienating the affected community.
Substance use among immigrants is a family problem (men are reported to abuse alcohol more than women; youth believe alcohol and marijuana use is expected in Canada).	• Create culturally safe support groups separated by gender
	• Family oriented approach to be applied in substance use prevention and treatment interventions
	• Help families identify and navigate risk factors for SUD-stress, mental health issues, acculturation.
	• Educate families on early signs of substance use
	• Build capacity for families to engage with loved ones on substance use issues
Lack of knowledge of substance use and addiction, its risks, and impacts are a major concern	• Create addiction content in the settlement services provided, such as English lessons
	• Engage settlement workers that have a pre-existing relationship with the communities in addressing substance use issues with the respective community
	• Organize knowledge-sharing events such as workshops on stigma reduction
	• Include substance use content for all immigrants seeking services from the settlement agencies
	• Amplify substance use and addiction problems immigrants have given the lack of race-based data on addiction.
Religious and cultural expectations of substance use disorders create barriers to care and cause stigma and shame	• Engage faith-based organizations affiliated with the target community in substance use prevention interventions
	• Build capacity among ethnic leaders to be actively involved in the community process
	• Recruit trusted community leaders to provide support to those affected by addiction
	• Engaged people with lived experience in the community
	• Make the community take ownership of the substance use prevention interventions
Lack of specific programs to address substance use and addiction among immigrants	• Conduct an environmental scan to explore how other jurisdictions support immigrant parents’ substance use treatment.
	• Encourage health care providers to screen for substance use risks and disorders
	• Use existing resources such as detox and pain clinics to increase access, such as HIPPIE
	• Create space for affected individuals to engage and support each other.
	• Provide access to timely youth counseling
	• Seek federal funding to create programs for substance use prevention among immigrants

## The Grant Application

A post-knowledge-sharing meeting was held with select group members: researchers, health care and settlement providers, and community members to debrief and identify the way forward. During this meeting, it was agreed that the knowledge acquired from this project regarding substance use among the newcomer population warranted developing a grant application. This grant will further explore some of the issues faced by the immigrant community, help raise awareness, aim to reduce stigma and harm, and create culturally appropriate educational tools. To proceed with the grant application, we identified lay people with community connections, such as religious and cultural leaders and professionals, to join the project as co-applicants. We intend to engage them in accessing the communities they represent and build the capacity for them to be change agents. We included a settlement agency as a knowledge user and persons with lived experiences per the grant application requirement.

A team comprising researchers, health care providers, community members from different ethnic groups, and people with lived experience was invited to join the team. A proposal titled *“Exploring substance use and addiction issues and building solutions among immigrants in Regina, Saskatchewan,”* was developed by this team. This proposal aimed to explore the immigrant community’s understanding of culturally safe approaches to increase self-efficacy and group consciousness and proactively address substance use risks in their communities.

The following research questions will guide this project:

a. What is the current state of substance use among immigrant communities in Regina?
b. How can immigrant stakeholders be best engaged to address the stigma of addiction and increase service utilization?
c. How can the immigrant stakeholders be best supported to create awareness and improve the responsiveness of addiction services for immigrants in Regina?

This study, funded by the Saskatchewan Health Research Foundation (SHRF), will adopt a collaborative process that ensures that the affected community is actively involved in all aspects of the research; this is known as the community-based participatory research (CBPR) approach. Patient-oriented research, which is based on the understanding that people with lived experiences have a good idea of their needs, will be the principle which guides this project.^37^

## Discussion

Canada is a multicultural country comprised of people of different nationalities and Indigenous Peoples, a population which can be further subcategorized as First Nation, Metis, and Inuit. The migration rate has increased in recent years and has contributed to the nation’s wealth and culture, generating a multifaceted populace with a global perspective.^38-40^ Considering the diversity of the populations within Canada, fostering mutual support and solidarity across communities is crucial for successfully integrating the immigrant population. One way to do so is to attend to the determinants of health pertinent to various communities.^41,42^ Understanding what immigrant communities require is essential to integrating immigrants into new populations successfully. It can be achieved through a vibrant community engagement process.

Although Canada does not collect race-based data on the health status of its population, evidence abounds regarding the health disparity that immigrants face. For instance, immigrants were disproportionately affected by COVID-19 compared to non-immigrant populations in Canada.^43^ Unlike non-immigrants, immigrants in Canada are more likely to work in jobs that have high-hazard exposure such as the case of COVID-19 and are likely to be employed in low-paying, labor-intensive jobs compared to non-immigrants.^44^ Such jobs tend to have limited social safety nets and support systems. Generally, immigrants have a high rate of poverty compared to non-immigrants and 31.4% of those that arrived between 2011 and 2016 live in poverty compared to 12.5% of non-immigrants.^45^

Since income is the most important determinant of health,^46^ immigrants are understandably likely to experience worse health outcomes compared to non-immigrants. Other DoHs that immigrants are likely to contend with are income and social status, employment and working conditions, physical environments, social support and coping skills, health behaviors, and access to health services. The health advantage that immigrants experiences at the time of admission due to the selective medical admissibility policy of barring potential immigrants who have conditions that would be a threat to the public health of Canadians or likely to have a higher burden to the health care system are not sustained as a result. Moreover, poverty and other DoHs are likely to exert immense mental stress which increases the risk for mental illnesses and the use of substance use as a maladaptive coping mechanism.

In this project, we have elected to focus on substance use and addiction within immigrant populations in Regina, Saskatchewan, based on the input from community partners that provide care and services to immigrants and observation by healthcare providers serving immigrants in Regina. A cursory review of Saskatchewan’s mental health and addiction action plan^36^ revealed that there are no explicit policies on substance use prevention for immigrants in the province. This may be due to a lack of empirical evidence to support relevant policy and program development. The community engagement process with diverse stakeholders that provide services to immigrants brought out important issues that helped understand discourses on substance use among immigrants in Regina Saskatchewan. These include pre-immigration risks shaped by substance use practices in the home country, experiences of adversities such as displacements and post-immigration factors such as settlement stress, family influences such as family functioning, parent-child dyad, parenting styles,^47^ cultural displacement, and availability and acceptability of substances of abuse.

Complexities surrounding risks for substance use are now well understood from a Canadian context. Therefore, to respond effectively to substance use in immigrants, practitioners and policymakers must employ an intersectional viewpoint, share power, and develop coalitions of common interest to create a safe space for the issues relevant to substance use, prevention, and treatment to be discussed as needed.^39^ Stakeholders can gain a greater understanding of the experiences that immigrants face through active engagement in research endeavors which can inform culturally responsive and inclusive programs that build resilience and prevent substance use. Hence they can help address the stigma of substance use and assist settlement agencies in delivering programs that include substance use prevention. In so doing, immigrants will be assisted to better integrate into their new country.^48-50^

Empowering immigrants with knowledge of substance use and available services provides them with a foundation that will allow them to control their lives better and potentially mitigate their risk for substance use. Recognizing factors such as trauma and adversity as potential triggers for substance use is vital when determining when to seek treatment and solutions before the emergence of unhealthy coping and addiction.^51-53^ One potential method of achieving this is by collaborating with health care providers to screen for substance use risks and pre-existing substance use disorders, not only at the time of immigration but in the subsequent years and by providing information in languages accessible to immigrants.

Developing an increased capacity for community partners to translate the available materials into ethnic languages can help overcome this barrier and make programs more accessible. In collaboration with key community gatekeepers (ie, clergy, community leaders, opinion leaders, and other influential positions), these community partners can collaborate to create culturally safe translation materials that enhance the knowledge of substance use, prevention, and treatment in immigrant communities.

Screening for risk of substance use among immigrants should be a routine standard practice after migration. Although this clinical practice would require culturally safe training skills, it will underscore the healthcare providers’ recognition of the risks of substance use that immigrants may face. Provider-initiated conversations with immigrants about substance use will assist in breaking the stigma and silence that surrounds substance use. Immigrant clients can thus find allies in the health settings to confide to and receive the help they need to prevent and support recovery from a substance use disorder. It will also respond to the needs identified in the Saskatchewan 10-year mental health and addiction strategy aimed at responding to diversities.^36^

## Conclusion

After the community engagement process and a cursory review of the literature, it has become evident that research focusing on understanding the experiences, perspectives, and interventions on substance use among immigrants in Canada is limited. Given that immigrants are culturally heterogeneous, a culturally informed approach must be employed to empower the immigrant community to engage in discourses on substance use and addiction. This focus is necessary for a Canadian context, especially considering the significant influence that immigration has on the country’s social and economic prosperity.

In addition, it is critical to support the advancement of immigrants and their families, who experience multiple determinants of health and are marginalized on multiple levels, to create a safe transition and settlement in Canada. Given the prevalence of diverse substances in Canada, practitioners and policymakers should anticipate difficulties immigrants may encounter in substance use prevention, reducing addiction stigma, increasing access to services, and reducing harm. Therefore, action to support them navigate the substance use prevention landscape is urgently needed.

Community-based research approaches empower tools to find common ground for priority identification across diverse immigrant populations. Thus, we can organically and iteratively build capacity for immigrants’ active involvement in addressing issues germane to them. Over time, selected community representatives can be empowered to advocate on behalf of their communities to increase self-efficacy, increase group conscientiousness, reduce stigma, and create space for vocalizing one’s concerns.

Organizing this event created a safe space to talk about an issue that although not visible compared to non-immigrant Canadians, yet has a negative impact on the community. This event provided an opportunity for diverse voices to be heard including those with lived experiences and practitioners in the settlement industry. In the absence of relevant statistics on the rates of substance use among immigrants in the province, the perspectives of the participants validated the concern that was observed in clinical practice.

Typical community engagement events are organized in person. However, due to the COVID-19 public health restrictions, the event was held virtually. This mode of consultation may have inadvertently limited the participation of important stakeholders, especially those with lived experiences and who may have challenges utilizing this technology. Moreover, virtual events limit the ability to observe nuanced nonverbal messages from the participants.

We appreciated that the immigrant population is heterogeneous and as such, it is impossible to have a satisfactory representation based on the geography of origin, cultural, or ethnic factors. Through this process, we learned that some ethnic groups are ready to engage on this topic, whereas others need more time to be primed for it and determine who in the community is best placed to be present in the conversation. This variation may be due to the perceived risks for substance use varying across immigrant groups and the importance attached to this issue.
